# Challenges and Complications in Treating Total Knee Arthroplasty in Morbidly Obese Patients

**DOI:** 10.7759/cureus.71433

**Published:** 2024-10-14

**Authors:** Bogdan A Bocea, Mihai D Roman, Radu S Fleaca, Nicolas C Ion, Radu Necula, Alexandru F Diconi, Romeo G Mihaila

**Affiliations:** 1 Biophysics, Lucian Blaga University, Sibiu, ROU; 2 Orthopedics and Traumatology, County Clinical Emergency Hospital, Sibiu, ROU; 3 Orthopedics and Traumatology, Lucian Blaga University, Sibiu, ROU; 4 Anatomy, Lucian Blaga University, Sibiu, ROU; 5 Orthopedics and Traumatology, Transilvania University, Brasov, ROU; 6 Hematology, Lucian Blaga University, Sibiu, ROU; 7 Hematology, County Clinical Emergency Hospital, Sibiu, ROU

**Keywords:** bmi, complication, infection, morbidly obese, tka, total knee arthroplasty

## Abstract

The number of total hip and total knee replacements has increased steadily each year. Today, they are routinely performed all over the world. The increasing prevalence of obesity has led to an increase in the number of total knee arthroplasties (TKAs) performed in patients with a higher body mass index (BMI) and also has a higher rate of complications. Possible complications are infection, instability, misalignment, osteolysis, or fracture. Revision arthroplasty can be challenging due to bone defects, ligament instability, and difficulty in fixation. We present the case of a 66-year-old morbidly obese patient who underwent TKA. After the operation, infection and a mechanical complication of periprosthetic fracture of the tibial component occurred. The infection made the treatment more difficult. The patient had to undergo several surgeries in several stages to address the post-surgical complications. TKA in obese patients leads to a higher risk of postoperative complications.

## Introduction

In 2009, more than one million total hip and knee arthroplasties were performed in the United States [1]. Total knee arthroplasty (TKA) is a well-established and successful treatment option for patients with end-stage knee osteoarthritis. It provides higher patient satisfaction with significantly improved quality of life [2].

Periprosthetic infections (PJIs) after a total knee replacement or total hip replacement (THA) implantation represent one of the most frequent and most fearful complications, with important consequences from the point of view of the patient's quality of life and social impact [3]. Established risk factors for PJI include elevated body mass index (BMI), diabetes mellitus, urinary tract infections, allogenic blood transfusion, and rheumatoid arthritis [4].

Studies have shown that obese patients have a higher rate of complications [5]. PJI and superficial wound infection are complications encountered more frequently in obese patients [6-8]. Other possible complications include instability, misalignment, osteolysis, fracture, and poor range of motion [9-11]. Deep vein thrombosis (DVT) is also a complication that occurs more in obese patients; in one of our studies, the single patient who developed DVT had the highest BMI of the lot [12].

## Case presentation

**Figure 1:**
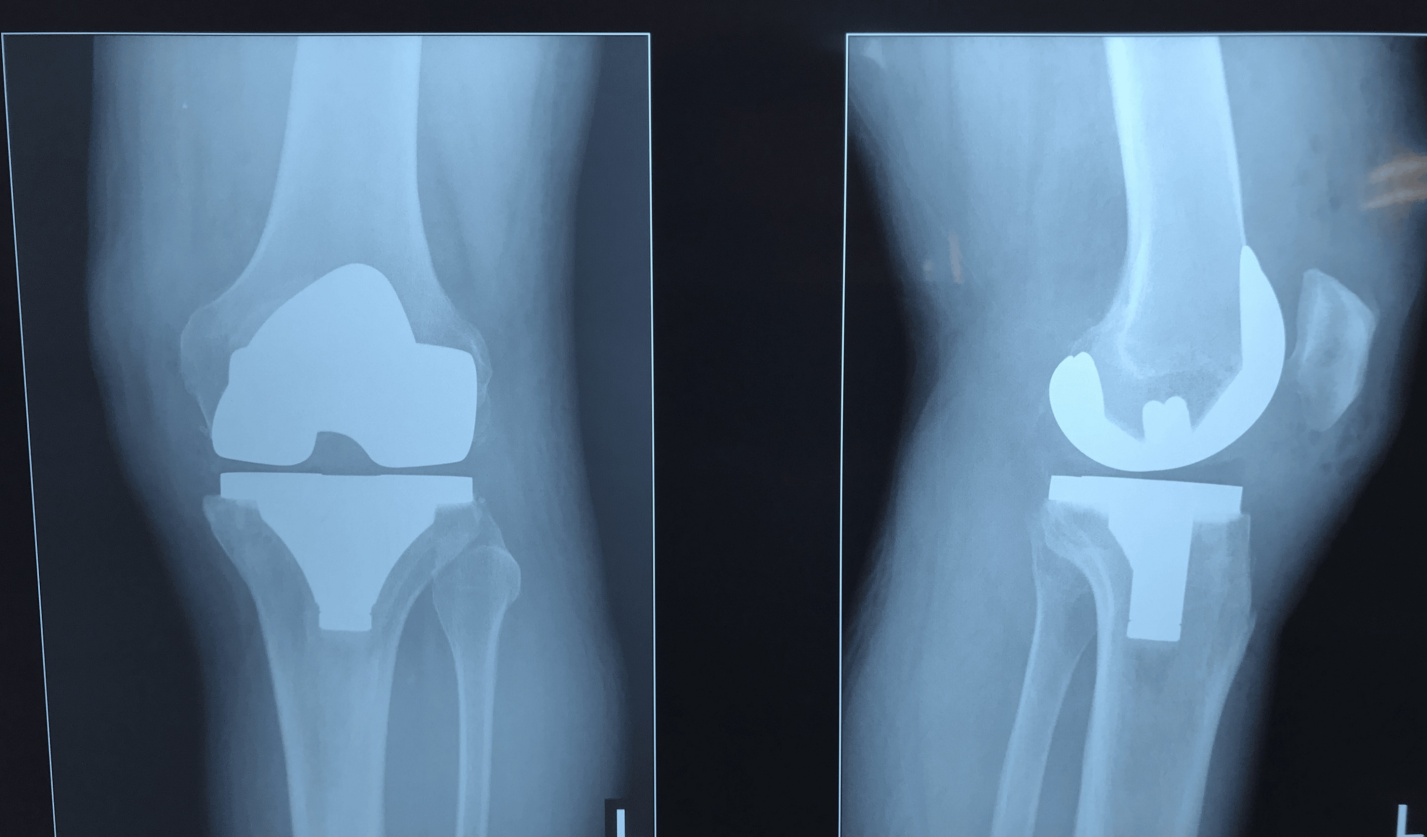
Postoperative radiograph 24 h after TKA surgery TKA: total knee arthroplasty

We present a case of a 66-year-old female patient with a BMI of 46.71 (weight: 121 kg, height: 1.61m). From personal pathological history, we note that the patient was only treated for hypertension. In November 2016, the patient underwent TKA (Figure 1). Before the surgery, the patient received 1 dose of 0.6ml of enoxaparin, and after the surgery, the patient received 0.6ml of enoxaparin, metamizole, chlorzoxazone, and ceftriaxone of 1g every 12h for 72h.

Postoperatively, from day one, the patient presented with a serosanguineous discharge, which we considered normal; however, it led us to resume prophylactic antibiotic treatment on day six with 1g of ceftriaxone every 12h. After 12 days postoperatively, we decided it was time to perform a bacteriological examination of the wound, which showed no pathogenic germs.

Twenty days postoperation, an arthroscopic lavage, debridement, and both chemical and mechanical cleansing were performed. A bacteriological examination was performed again, revealing an Enterococcus infection treated with ceftriaxone until discharge. The local evolution was favorable with the healing of the operative wound. The patient did not return for the recommended check-ups at three, six, and 12 months.

**Figure 2:**
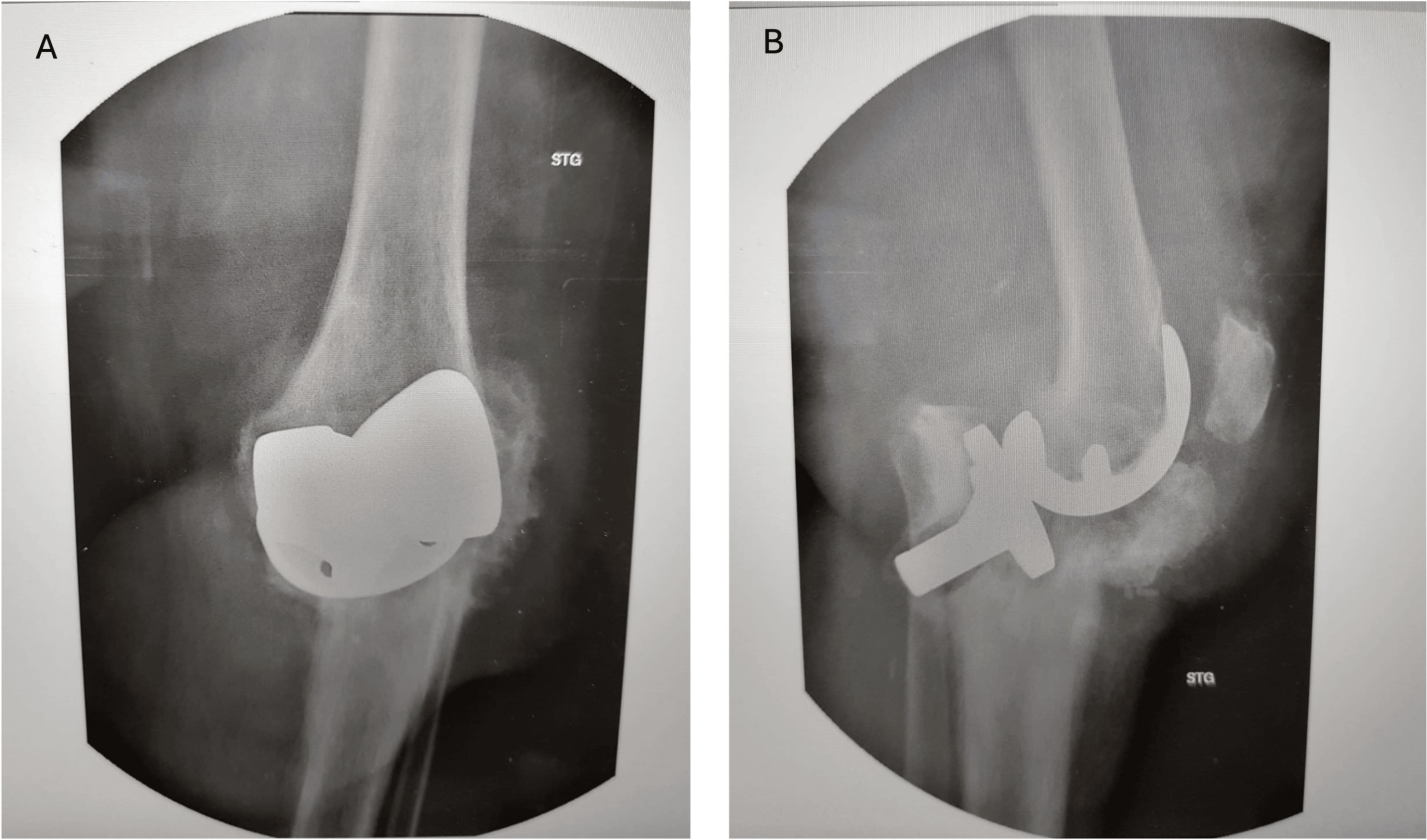
X-ray taken at the time of admission. Periprosthetic fracture of the tibial component and a tibial tuberosity fracture with displacement A: Antero-posterior radiograph of the periprosthetic fracture; B: Lateral radiography of the periprosthetic fracture

One and a half years after the surgery, the patient had pain in the operated knee but presented to the medical rehabilitation ward after 2 years. A control X-ray was performed, revealing a periprosthetic fracture with tibial compression and tilting, as well as a large displaced fracture of the tibial tuberosity. The patient was transferred to the orthopedic department for specialized treatment (Figure 2) [13].

**Figure 3:**
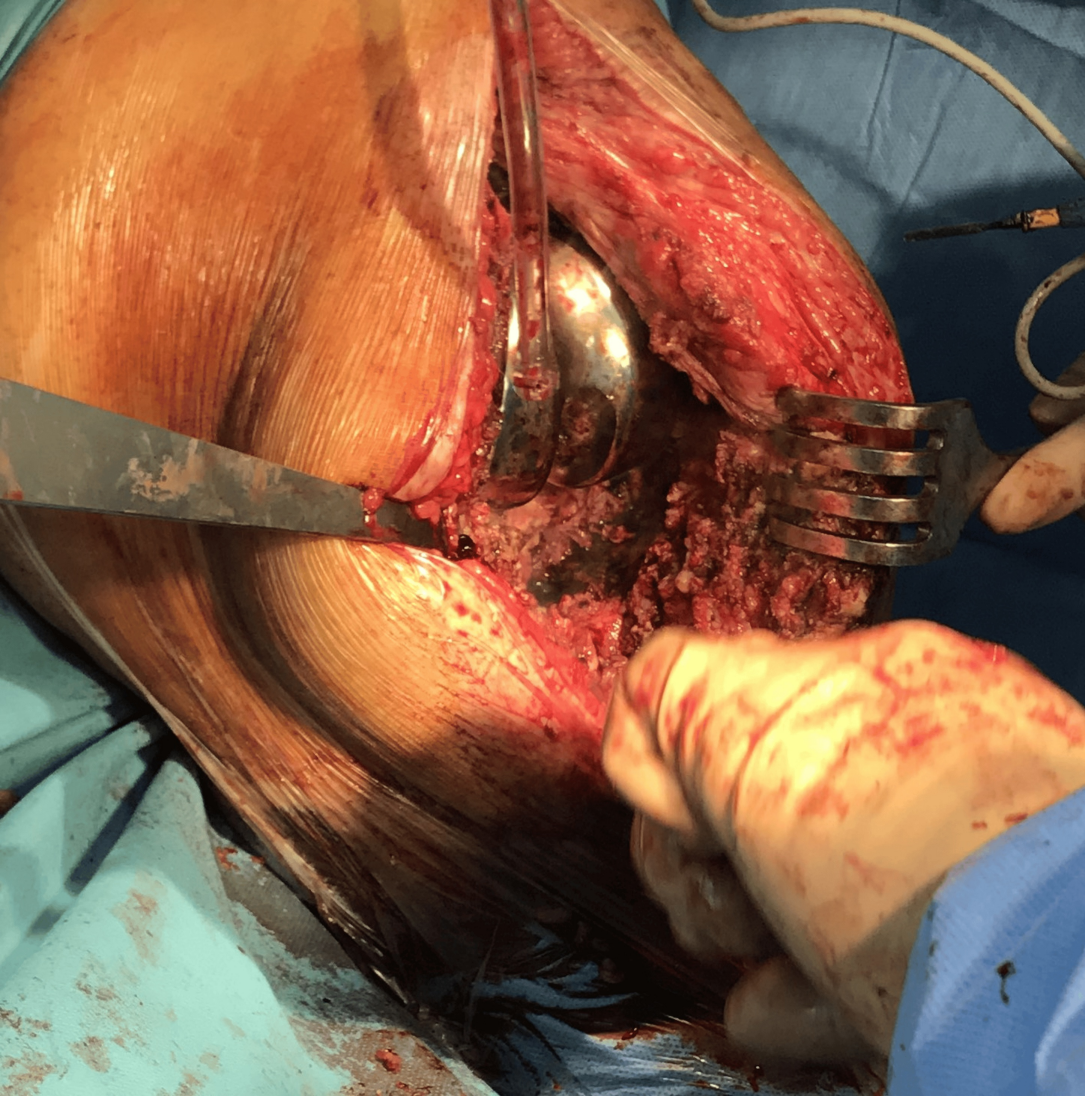
The tibial tuberosity fracture with friable and avascular bone.

**Figure 4:**
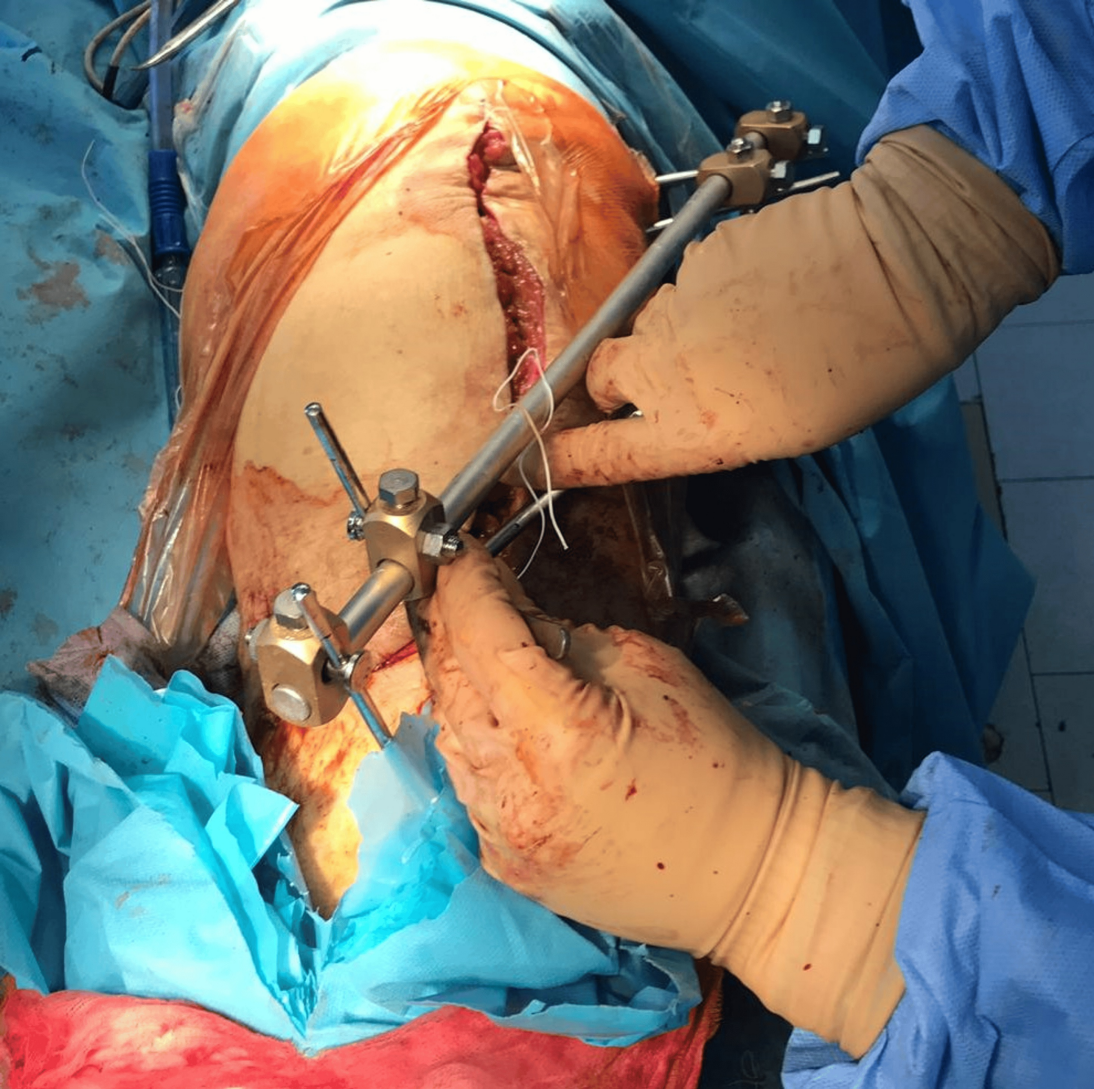
The external fixator that was applied to stabilize the knee.

**Figure 5:**
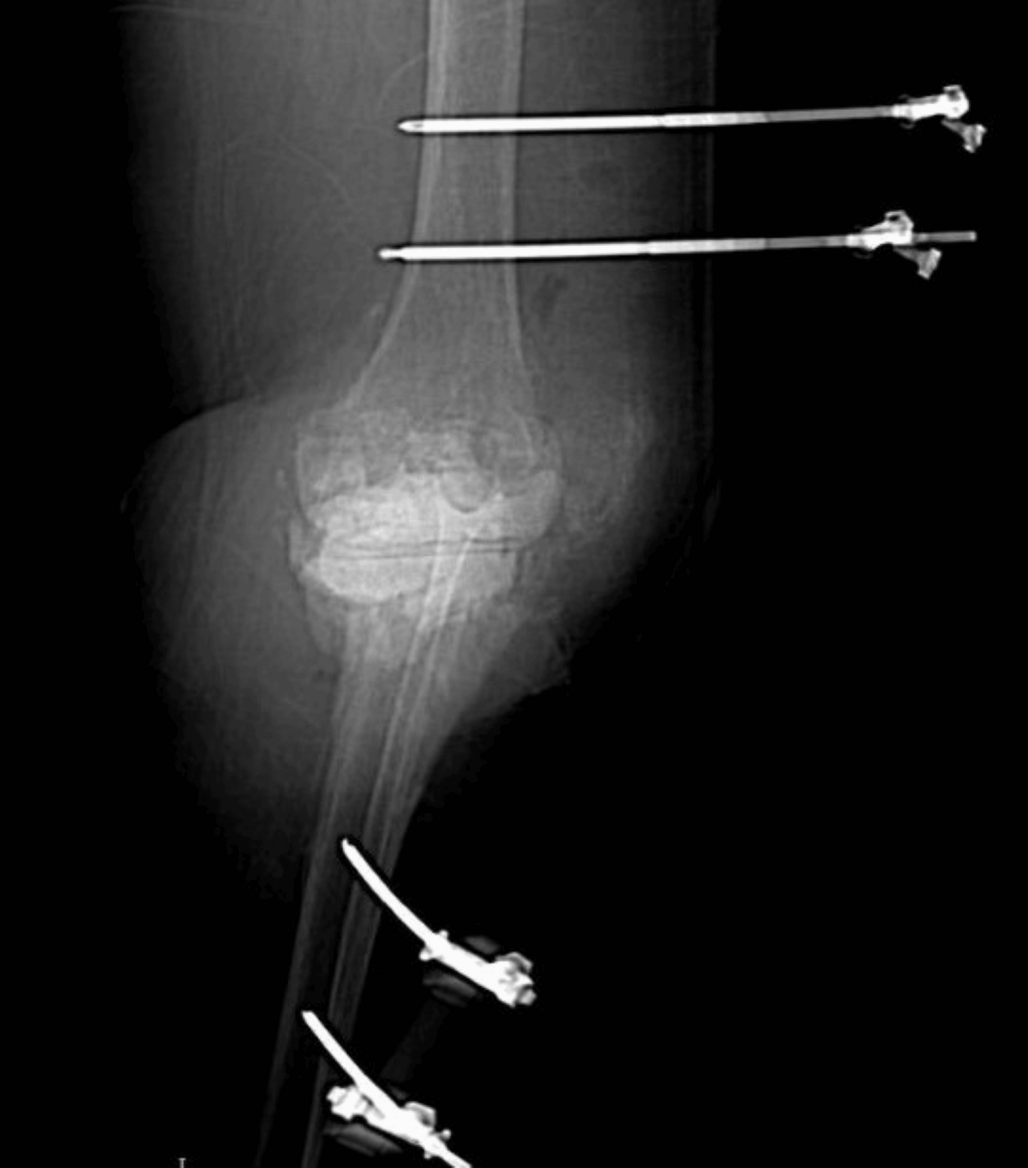
Postoperative control radiograph.

Laboratory tests reveal increased inflammatory samples (fibrinogen- 681mg/dL; C-reactive protein (CRP)- 45mg/dL; erythrocyte sedimentation rate (ESR)- 91mm/hr). Local examination shows signs of infection (tumor, flushing, heat, and pain). The therapeutic indication is a two-stage revision. The prosthesis is extracted, the femoral component is well fixed, and the tibial component is gently removed. Anterior tibial tuberosity fracture is examined, where friable, avascular bone is identified and removed. An antibiotic spacer is fitted. Ligamentous stability is checked, and a decision is made to mount an external fixator (Fig 3-5).

Six knee areas were bacteriologically examined during the operation, and a Proteus and Staphylococcus aureus infection was found. We administered Ceftazidimum 1grx2fl/day for 14 days during hospitalization and four weeks of Levofloxacinum 500mg 2tab/day and Sulfamethoxazole + Trimethoprimum 400mg/80mg 2x2tab/day at home.

**Figure 6:**
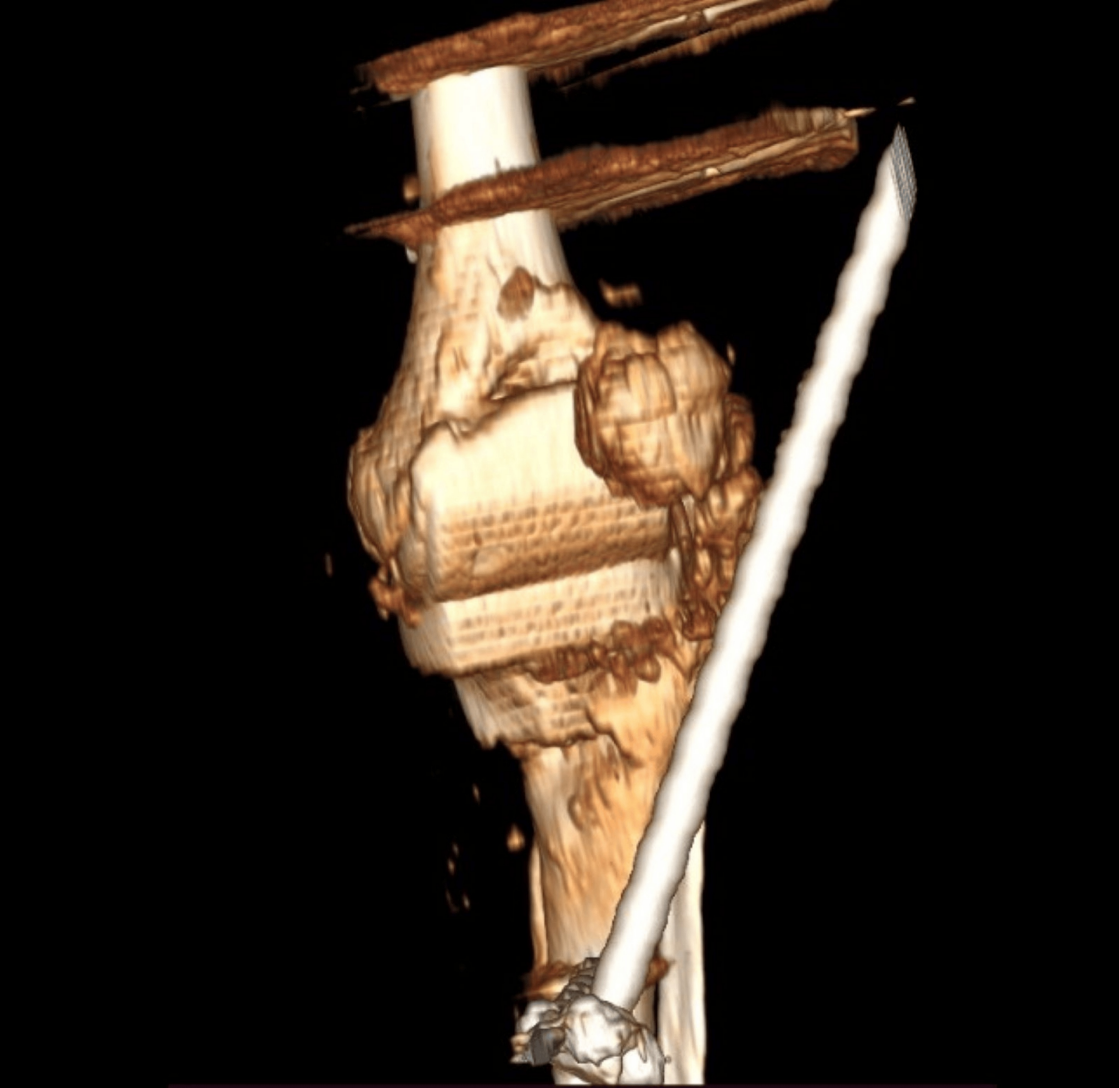
Knee CT with Spacer and external fixator mounted.

A CT scan was performed every three months, and tests were performed to determine the next therapeutic course (Fig 6). The external fixator was removed, and the clinical examination revealed another ligament instability, so the patient could not perform knee extension. A decrease in inflammatory samples was observed, but not within normal limits (fibrinogen- 410mg/dL; CRP- 6.6mg/dL; ESR- 26mm/hr).

**Figure 7:**
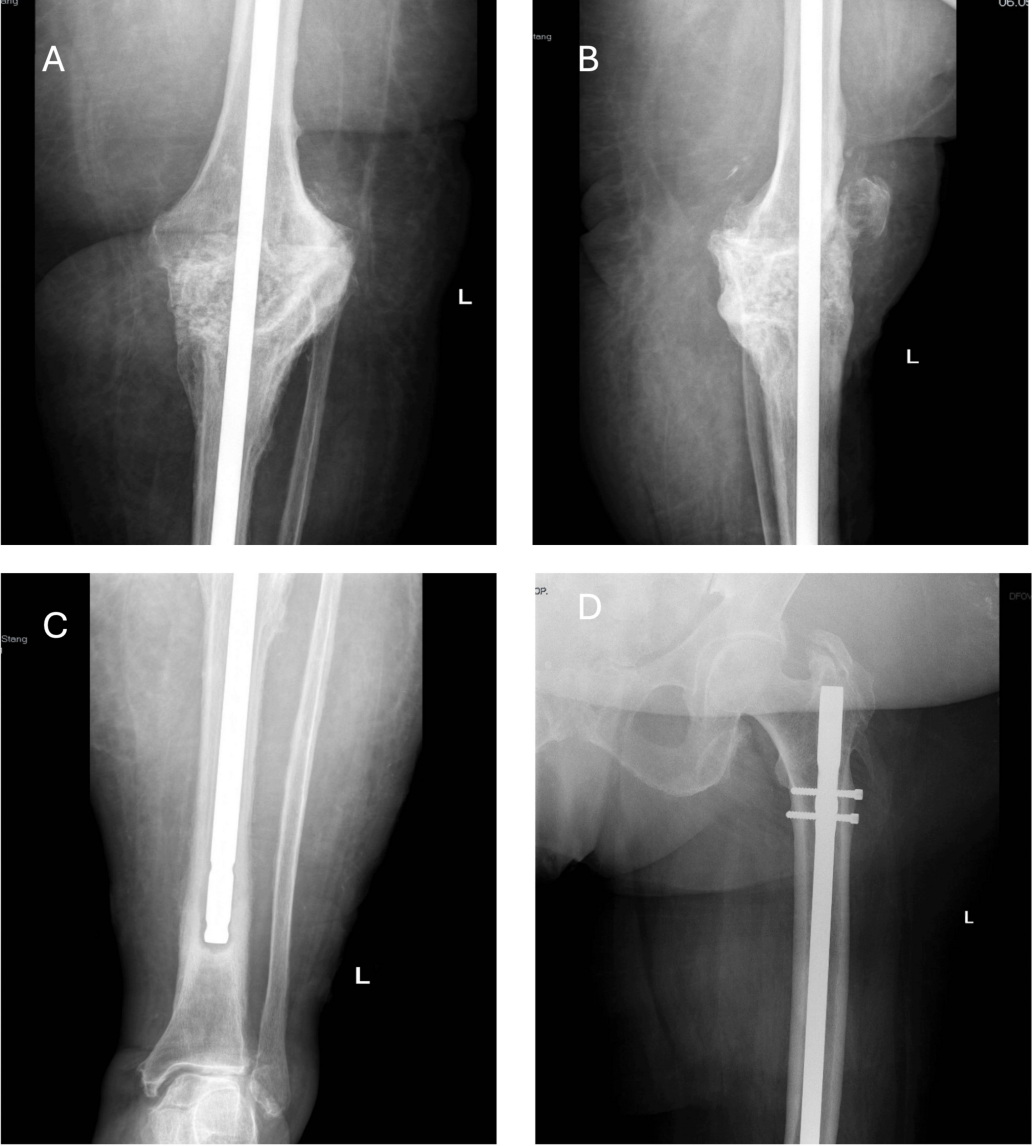
Centromedullary knee arthrodesis nail, fixed proximally with two screws A - Antero-posterior radiograph of the knee with CM rod and bone graft: B: Antero-posterior radiograph of the knee with CM rod; C: Antero-posterior radiograph of the CM rod in the tibia; D: Antero-posterior radiograph of the CM rod fixed in the femur with 2 screws

Six months after step one of revision, the inflammatory markers normalized (fibrinogen- 389mg/dL; CRP- 2.99mg/dL; ESR- 19mm/hr), and the local examination was normal. Due to the lack of tibial tuberosity and the ligamental instability, the decision was made to perform an arthrodesis with a CM rod (Charfix system - Intramedullary osteosynthesis of Tibia - Lewickie 3B, 16-061 Juchnowiec Kościelny, Poland), and to fill the bone defect with a bone graft prepared from the bone bank (Fig 7). The CM was specially ordered and measured in advance to get equal length in both lower limbs. The surgery was completed without complications. Postoperative evolution was favorable. The patient did not return for the recommended check-ups (3, 6, and 12 months).

**Figure 8:**
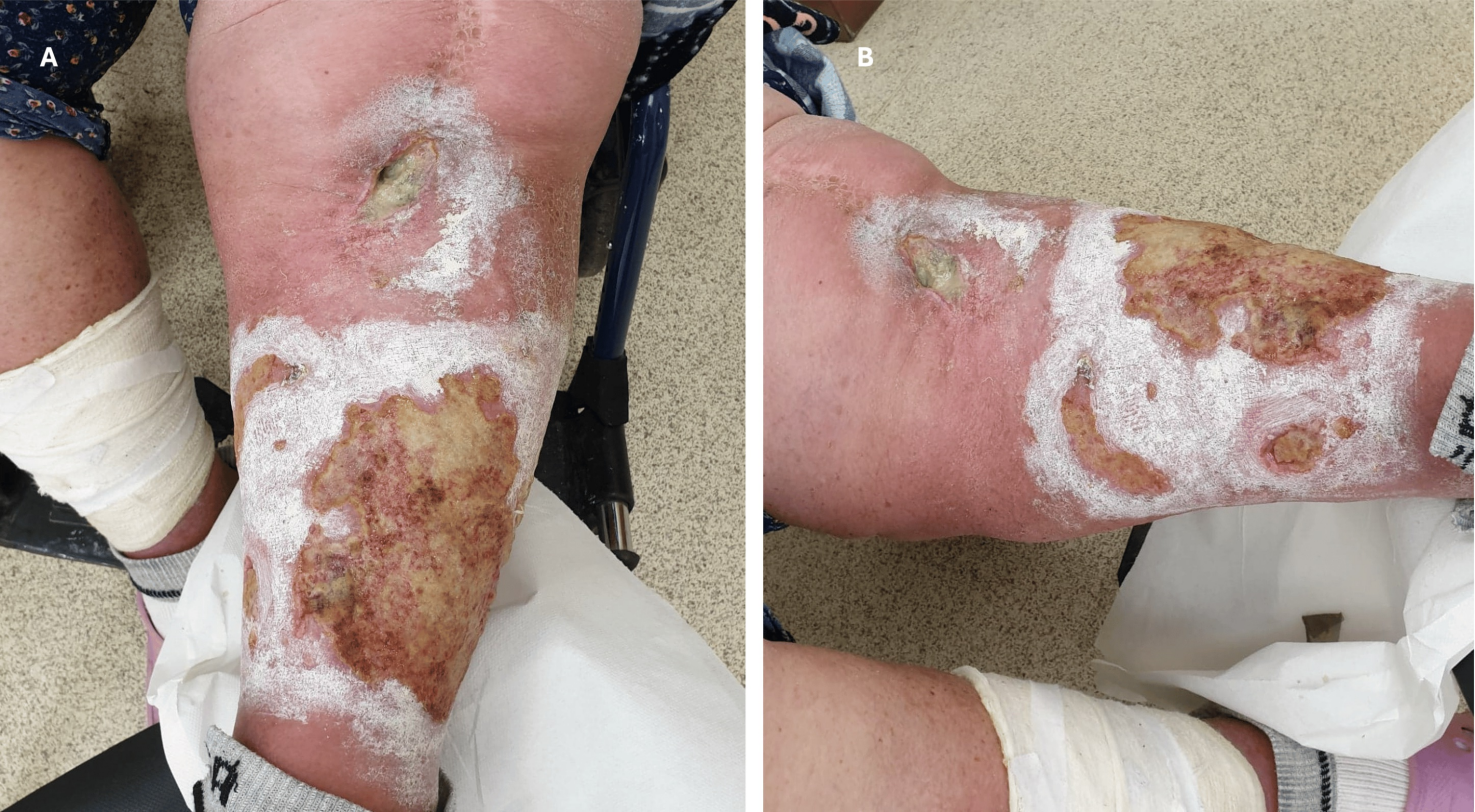
Extensive venous ulcers and knee fistula. A simple chemical and mechanical examination shows that this fistula communicates with the "knee joint" A: Knee fistula and venous ulcers of the lateral part of the leg; B: Extensive venous ulcers of the medial part of the leg

Three years after knee arthrodesis, the patient presents to the outpatient clinic with superinfected varicose ulcers and a fistula in the knee communicating with the arthrodesis area (Fig 8). Last year, the patient visited a dermatologist and a general surgery consultant for varicose ulcers. They treated these ulcers and recommended she see an orthopedic specialist, but she did not comply with their recommendation.

The bacteriological examination was collected and revealed a Morganella morganii infection. The patient refused admission and was given home treatment with Ceftazidimum 1grx2fl/day for 21 days without success.

**Figure 9:**
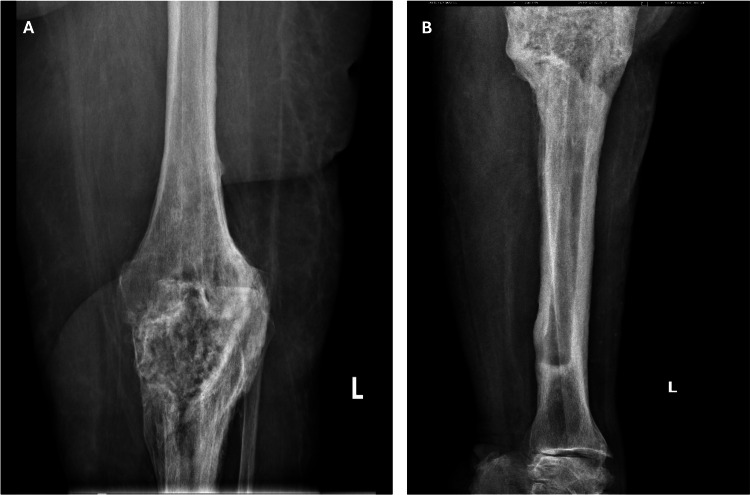
Postoperative radiographs after removal of the arthrodesis nail A: Antero-posterior radiograph of the knee after CM rod removal; B: Antero-posterior radiograph of the knee and tibia after CM rod removal

After six months, she was admitted to the orthopedic ward. On admission, a bacteriological examination with an antibiogram was again taken, reconfirming Morganella infection. Inflammatory samples are elevated (CRP- 49.6mg/dL; Fibrinogen- 668.4mg/dL; and ESR- 76mm/hr). The arthrodesis rod was removed, debridement of the infected bone, chemical and mechanical cleaning was performed, and antibiotic treatment was continued (Fig 9). In the first phase, treatment with Ceftazidimum 1grX2fl/day for two weeks is started without success, and then Amikacinum 500mg/2gr 2fl/day is added for another two weeks. A bacteriological examination was done during this period, and an antibiogram was performed weekly. At the antibiogram four weeks after admission, the germ had become resistant to Ceftazidinum. Ceftazidinum was replaced with Meropenem 1gr/day, and the treatment was continued for two months without success. Then Piperacillinum + Tazobactamum 4g/0.5g 2fl/day and Amikacinum 500mg/2ml 2fl/day for one week, then replaced with Piperacillinum + Tazobactamum with Piperacillinum 4g/0.5g for two weeks. All antibiotic therapy was administered on an antibiogram basis after consultation with the infectious diseases physician.

**Figure 10:**
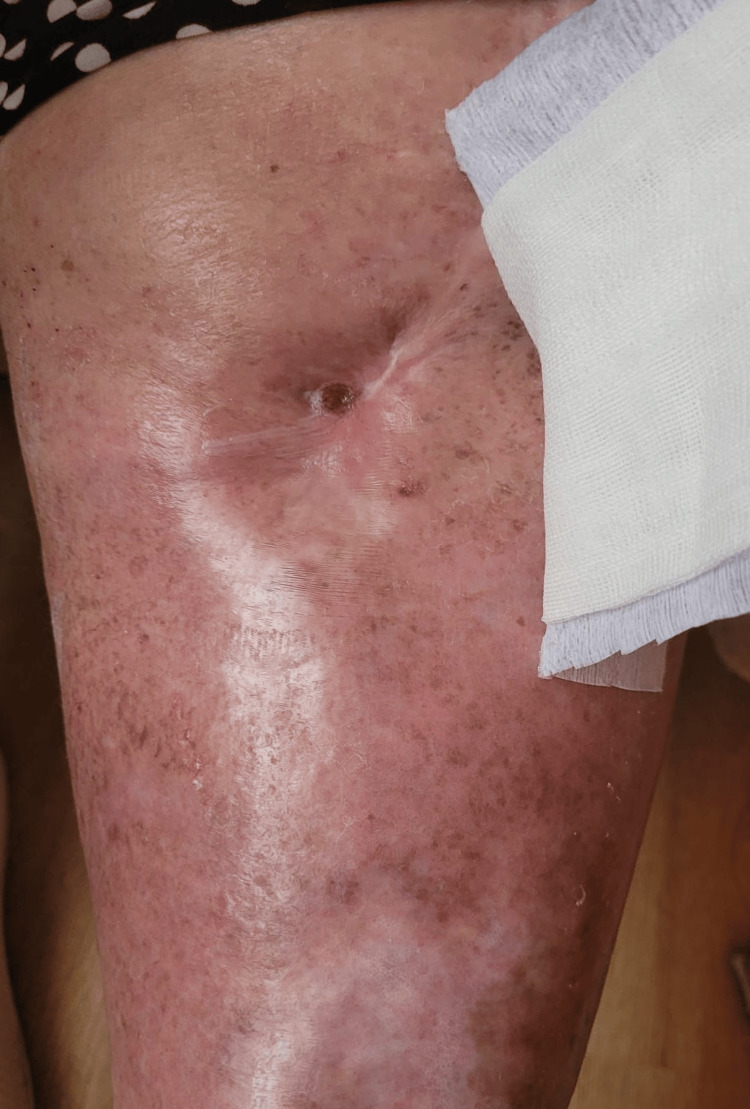
Varicose ulcers are healed, and the fistula is closed

The wound closed after antibiotic treatment and repeated chemical and mechanical toileting (Fig 10). At discharge, inflammatory samples were still above normal: CRP of 14.1mg/dL, fibrinogen of 558.0mg/dL, and ESR of 92mm/hr. The patient was discharged with a healed wound.

## Discussion

Periprosthetic fracture after primary TKA was reported to reach up to 5.5%. Some authors suggest a lower incidence of tibial PPF after primary TKA compared to the femoral side [14]. These fractures could be more common in patients with a high BMI, female gender, and a tendency for smaller tibial sizes [15]. Our patient had a BMI of 46.71 and received a small tibial size component.

The fact that our patient had very few complaints made the case more difficult two times. After feeling pain in the knee, she waited six months before her presentation to a therapist. That led to a tibial tuberosity fracture with friable and avascular bone. Studies suggest that in such a periprosthetic fracture, osteosynthesis is indicated with a revision of the tibial component [16]. In our case, we couldn't save the greater tuberosity. Combined with the infection, we lost a lot of bone stock from the proximal tibial, which led to a very unstable knee. Taking all this into consideration, we decided on a knee arthrodesis with a centromedullary nail fixed proximally with two screws.

Studies from the literature confirm that there is a higher risk of PJI in patients with high BMI [17]. The fact that the patient had few complaints also made treating the infection more difficult. When the patient agreed to be admitted to the orthopedic ward, the infection had extended to the entire knee region with significant bone involvement. The methicillin-resistant Staphylococcus aureus (MRSA)-identified bacteria, Morganella morganii, is a relatively rare isolated bacteria in PJI, making it hard to treat [18].

## Conclusions

In morbidly obese patients, the frequency of complications is much higher after TKA than in patients with a normal BMI. In patients with very low compliance, the treatment becomes more demanding and difficult.
